# Utilizing Remote Real-Time Videoconferencing to Expand Access to Cancer Genetic Services in Community Practices: A Multicenter Feasibility Study

**DOI:** 10.2196/jmir.4564

**Published:** 2016-02-01

**Authors:** Angela Bradbury, Linda Patrick-Miller, Diana Harris, Evelyn Stevens, Brian Egleston, Kyle Smith, Rebecca Mueller, Amanda Brandt, Jill Stopfer, Shea Rauch, Andrea Forman, Rebecca Kim, Dominique Fetzer, Linda Fleisher, Mary Daly, Susan Domchek

**Affiliations:** ^1^ Division of Hematology-Oncology Department of Medicine The University of Pennsylvania Philadelphia, PA United States; ^2^ Abramson Cancer Center The University of Pennsylvania Philadelphia, PA United States; ^3^ Department of Medical Ethics and Health Policy Philadelphia, PA United States; ^4^ Division of Hematology-Oncology Department of Medicine The University of Chicago Chicago, IL United States; ^5^ Center for Clinical Cancer Genetics and Global Health The University of Chicago Chicago, IL United States; ^6^ Biostatistics and Bioinformatics Facility Fox Chase Cancer Center Temple University Health System Philadelphia, PA United States; ^7^ Department of Clinical Genetics Fox Chase Cancer Center Temple University Health System Philadelphia, PA United States; ^8^ Center for Injury Research and Prevention The Children’s Hospital of Philadelphia Philadelphia, PA United States; ^9^ Basser Research Center Abramson Cancer Center The University of Pennsylvania Philadelphia, PA United States

**Keywords:** health care delivery, dissemination and implementation, cancer genetics, genetic counseling, genetic testing, telemedicine

## Abstract

**Background:**

Videoconferencing has been used to expand medical services to low-access populations and could increase access to genetic services at community sites where in-person visits with genetic providers are not available.

**Objective:**

To evaluate the feasibility of, patient feedback of, and cognitive and affective responses to remote two-way videoconferencing (RVC) telegenetic services at multiple sociodemographically diverse community practices without access to genetic providers.

**Methods:**

Patients at 3 community sites in 2 US states outside the host center completed RVC pretest (visit 1, V1) and post-test (visit 2, V2) genetic counseling for cancer susceptibility. Surveys evaluated patient experiences, knowledge, satisfaction with telegenetic and cancer genetics services, anxiety, depression, and cancer worry.

**Results:**

A total of 82 out of 100 (82.0%) approached patients consented to RVC services. A total of 61 out of 82 patients (74%) completed pretest counseling and 41 out of 61 (67%) proceeded with testing and post-test counseling. A total of 4 out of 41 (10%) mutation carriers were identified: *BRCA2*, *MSH2*, and *PMS2*. Patients reported many advantages (eg, lower travel burden and convenience) and few disadvantages to RVC telegenetic services. Most patients reported feeling comfortable with the video camera—post-V1: 52/57 (91%); post-V2: 39/41 (95%)—and that their privacy was respected—post-V1: 56/57 (98%); post-V2: 40/41 (98%); however, some reported concerns that RVC might increase the risk of a confidentiality breach of their health information—post-V1: 14/57 (25%); post-V2: 12/41 (29%). While the majority of patients reported having no trouble seeing or hearing the genetic counselor—post-V1: 47/57 (82%); post-V2: 39/41 (95%)—51 out of 98 (52%) patients reported technical difficulties. Nonetheless, all patients reported being satisfied with genetic services. Compared to baseline, knowledge increased significantly after pretest counseling (+1.11 mean score, *P*=.005); satisfaction with telegenetic (+1.74 mean score, *P*=.02) and genetic services (+2.22 mean score, *P*=.001) increased after post-test counseling. General anxiety and depression decreased after pretest (-0.97 mean anxiety score, *P*=.003; -0.37 mean depression score, *P*=.046) and post-test counseling (-1.13 mean anxiety score, *P*=.003; -0.75 mean depression score, *P*=.01); state anxiety and cancer-specific worry did not significantly increase.

**Conclusions:**

Remote videoconferencing telegenetic services are feasible, identify genetic carriers in community practices, and are associated with high patient satisfaction and favorable cognitive and affective outcomes, suggesting an innovative delivery model for further study to improve access to genetic providers and services. Potential barriers to dissemination include technology costs, unclear billing and reimbursement, and state requirements for provider licensure.

## Introduction

*BRCA1/2* testing for predisposition to breast and ovarian cancer is one application of personalized medicine that has become standard practice in cancer prevention [1]. Access to cancer risk assessment and testing when appropriate is now required for the National Accreditation Program for Breast Centers [2]. Cancer genetics services have traditionally included in-person pretest and post-test (ie, result disclosure) counseling with an experienced provider [3]. Given a limited workforce of genetic providers who are generally located in academic and urban centers, this in-person delivery model often requires patients to travel to a potentially distant and unfamiliar medical setting to receive cancer genetic testing with a genetic provider. Some patients proceed with testing without a genetic provider (ie, with their local physician) or they do not proceed with testing at all [3,4]. Thus, there remain significant access, time, and patient cost barriers to in-person genetic services that could contribute to disparities in both uptake and outcomes of genetic services [5,6]. Equally important, genetic testing without genetic providers (ie, with one’s primary care physician or other nongenetics provider) has been associated with inappropriate testing and overtesting, which could increase health care costs [7,8]. Thus, as clinically relevant genetic applications increase, innovative delivery models to promote access to cancer genetics specialists are needed [9].

Remote two-way, real-time videoconferencing (RVC) has been increasingly utilized to provide educational, behavioral, and medical services [10,11]. In some areas (eg, education and supportive care, psychotherapy and psychiatric services, and remote monitoring or follow-up care in cardiac and respiratory diseases) there is strong evidence for benefits of remote care, such as RVC, as an alternative to in-person delivery [10,11]. In other areas, there is evidence that RVC is feasible and potentially valuable but further research is needed (eg, stroke rehabilitation, neurologic diseases, genetics, and diabetes care) [10,12-16]. Studies have demonstrated acceptability and feasibility of RVC for delivery of a wide range of medical services in underserved areas, including dermatology [17], stroke, pediatric subspecialties [18-20], obstetrics [21], endocrinology [22], psychiatry [23-27], and neurology [28,29]. Similarly, RVC has been utilized to provide genetic services (ie, telegenetics) to populations where geographic, socioeconomic, or provider factors have limited the use and dissemination of in-person genetic services [30-40]. Of these studies, many have demonstrated high patient satisfaction, but most have been relatively small and reported limited patient-reported outcomes [30,41]. None have been theoretically informed, and few have reported technology disruptions or challenges [40,42]. The largest study of RVC in cancer genetics compared patient experiences, including knowledge and distress, and reported no differences between in-person and RVC genetic services, although this was not a randomized study [12]. Additionally, the clinical geneticist was the provider utilizing RVC with a genetic counselor on site (ie, in-person) with the patient during the consultation. A recently published randomized trial of entirely RVC genetic services versus in-person services provided by a traveling genetic counselor in rural clinics reported no difference in patient satisfaction and lower costs with RVC, but poorer uptake in the RVC arm [40].

In this study, we sought to evaluate a resource-extending model by providing genetic services entirely remotely at community medical facilities with no options for in-person genetic services. In this model, the genetic provider is physically at the host center and services are provided entirely remotely in the patient’s local medical facility. Additionally, we utilized communication protocols informed by stakeholders (eg, patient and provider feedback) and all providers were trained for videoconferencing communication. Our primary aim was to evaluate the feasibility of using RVC to provide pre- and post-test counseling by a host center genetic counselor and to evaluate this model at multiple community sites. Second, we sought to evaluate a wide range of patient-reported outcomes, including qualitative advantages, disadvantages, and experiences. We also sought to evaluate cognitive (eg, knowledge) and affective responses (eg, anxiety, depression, cancer worry, and satisfaction) to RVC telegenetic services in geographically and sociodemographically diverse community medical practices.

## Methods

### Participants

Participants were recruited at 3 community medical sites in New Jersey (NJ) and Delaware (DE), USA, all sites without a genetic provider on staff (see Table 1).

**Table 1:** Participant characteristics.

Characteristic		Approached (n=100)	Completed V1^a^ (pretest counseling) (n=61)	Completed V2^b^ (test disclosure) (n=41)
Age in years, mean (SD, range)		54 (14, 23-87)	54 (13, 26-85)	56 (13, 28-85)
**Self-reported race/ethnicity, n (%)**				
	White	74 (74.0)	47 (77)^j^	33 (80)^j^
	African American/black	12 (12.0)	8 (13)	4 (10)
	Hispanic/Latino/other	14 (14.0)	6 (10)	4 (10)
Gender (female), n (%)		98 (98.0)	60 (98)	40 (98)
**Community site, n (%)**				
	Kennedy Health System (NJ)	26 (26.0)	14 (23)	7 (17)
	Community Medical Center (NJ)	47 (47.0)	29 (48)	17 (42)
	Bayhealth Medical Center (DE)	27 (27.0)	18 (30)^j^	17 (42)^j^
**Education** ^c^ **, n (%)**				
	High school or less	18/81 (22)	15 (25)	11 (27)
	Some college/associates	25/81 (31)	16 (26)	11 (27)
	College graduate	29/81 (36)	24 (39)	16 (39)
	Graduate or postgraduate	9/81 (11)	6 (10)	3 (7)
Marital status^d^ (married^e^), n (%)		49/80 (61)	39 (64)	25 (61)
Personal history of cancer^c^ (yes), n (%)		41/81 (51)	33 (54)^j^	28 (68)^j^
Known mutation in family^c^ (yes), n (%)		7/81 (9)	5 (8)	3 (7)
Number of FDRs^f^/SDRs^g^ with cancer, mean (SD)		N/A^h^	4.18 (2.74)	3.80 (2.62)
**Genetic testing, n (%)**				
	*BRCA1/2*	N/A	N/A	38 (93)
	Lynch syndrome	N/A	N/A	2 (5)
	Both	N/A	N/A	1 (2)
**Test result, n (%)**				
	Uninformative/negative	N/A	N/A	35 (85)
	Positive	N/A	N/A	4 (10)
	True negative	N/A	N/A	2 (5)
	VUS^i^	N/A	N/A	0 (0)

^a^V1: visit 1.

^b^V2: visit 2.

^c^Of the total approached participants, 19 were without available information.

^d^Of the total approached participants, 20 were without available information.

^e^Includes domestic partnership.

^f^FDR: first-degree relative.

^g^SDR: second-degree relative.

^h^N/A: not applicable.

^i^VUS: variant of uncertain significance.

^j^ *P*<.05.

Eligible participants were able to communicate in English, were over 20 years old, and were potential candidates for *BRCA1/2* or Lynch syndrome genetic testing as per National Comprehensive Cancer Network (NCCN) guidelines. Hearing-impaired patients were excluded from this study. The study was approved by the University of Pennsylvania (UPENN) Institutional Review Board (IRB); IRB authorization agreements were completed with each of the participating sites. Participants provided informed consent for study participation and were recruited between April 2013 and June 2014.

### Remote Videoconferencing Telegenetic Delivery Model

We adapted previously developed communication protocols for telephone delivery for the purpose of real-time, two-way RVC services [43,44]. Our initial RVC telegenetics protocol was piloted (April-August 2012) at the Fox Chase Cancer Center with a community practice in New Jersey. We utilized patient and provider feedback and review of videorecorded visits (n=10) to refine our protocol for this multicenter study.

RVC and technology support were provided through Mid-Atlantic Gigapop in Philadelphia for Internet 2 (MAGPI). The community sites’ and the host‘s (University of Pennsylvania) central processing units were outfitted with high-definition Web cameras with built-in microphones and Cisco videoconferencing software applications. All connections were at 768 kbps with a minimum connection speed of 384 kbps. Connections between sites were made with a Codian bridge utilizing Advanced Encryption Standard approaches for security.

Patients completed RVC pretest counseling visits with a genetic counselor who was at the University of Pennsylvania. Community clinical staff were available on-site during RVC study visits to assist patients with technology challenges, address questions, and facilitate clinical genetic testing. Patients who proceeded with testing were scheduled for RVC post-test counseling with a genetic counselor. A total of 26 out of 41 patients (63%) met with a community site physician to discuss medical recommendations at the time of the post-test counseling session with the genetic counselor. Others had medical follow-up separate from their post-test counseling session.

Similar to our other studies evaluating adaptations to traditional face-to-face counseling [43,44], we developed standardized counseling topic checklists—15 pretest and 12 post-test counseling topics. Other key components of the RVC telegenetic protocol included visual aids, standardized provider probes to evaluate patient understanding and emotional responses, and situational probes to address technology disruptions and other challenges specific to RVC. All board-certified genetic counselors (n=4) were licensed in outside states according to state laws and completed RVC telegenetic communication training, including a mock visit with individualized feedback from a clinical health psychologist with expertise in health communication, also one of the study authors (LPM). Genetic counselors completed pre- and post-test counseling checklists and all RVC telegenetic visits were recorded to assess fidelity to the protocol.

Counseling checklists revealed good fidelity to pretest (mean 83%) and post-test (mean 87%) counseling topics. A total of 20% of recorded visits were reviewed to ensure that provided completed counseling checklists reflected completion of the counseling topics. This audiotape fidelity review revealed very good consistency with provider-completed checklists (89%). Most discrepancies were clerical rather than counseling omissions.

### Outcome Variables

#### Overview

**Figure 1:**
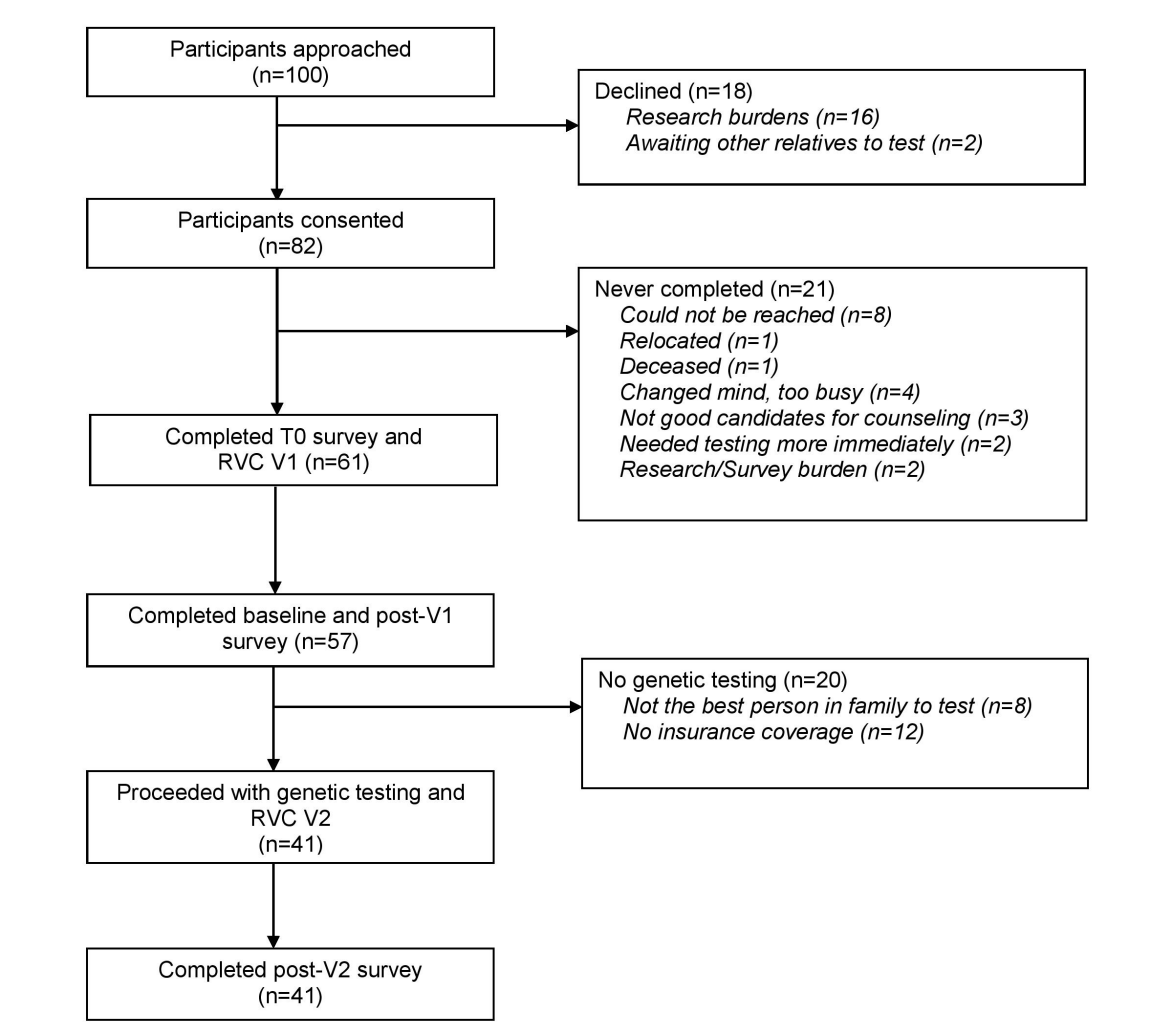
Study schema. RVC: remote videoconferencing; V1: visit 1, pretest counseling visit; V2: visit 2, test disclosure visit.

As the successful translation of personalized medicine into improvements in population health requires understanding behavioral change at patient, provider, and organizational levels, we employed our overarching conceptual model integrating the Self-Regulation Theory of Health Behavior and the Diffusion of Innovation Theory. The Diffusion of Innovation Theory has been successfully applied in numerous studies of systems adoption and implementation of innovative information technology [45-48]. The Self-Regulation Theory of Health Behavior [49] has been utilized in descriptive and intervention-based research of individuals’ responses to health threats, including genetic predisposition to disease. Our conceptualization of the Self-Regulation Theory of Health Behavior and the Diffusion of Innovation Theory informs the evaluation of the immediate (<72 hours) and delayed responses to our novel delivery model for genetic services [13]. Participants completed self-administered surveys online or by pen and paper at baseline (T0), and after pretest (T1) and post-test (T2) RVC telegenetic visits. Study data were collected and managed using Research Electronic Data Capture (REDCap) [50], a secure, Web-based application for data capture in research studies. All REDCap surveys were IRB approved, closed, and tested for usability, and they utilized adaptive questioning [51]. Completion rates are shown in Figure 1.

#### Opinions and Experiences With Real-Time Videoconferencing Telegenetics (T1, T2)

Open-ended items adapted from related research [43,44] were utilized to elicit patient experiences with, perceptions of, and suggestions for, improving RVC telegenetic visits.

#### Satisfaction With Genetic Services (T1, T2)

Satisfaction with genetic services (T1, T2) was measured with a 9-item scale evaluating satisfaction with health communication and utilized in related research [52,53] (Cronbach alpha=.80).

#### Satisfaction With Telemedicine Delivery (T1, T2)

Satisfaction with telemedicine delivery (T1, T2) was assessed with 13 items adapted for genetic counseling to evaluate patient-perceived provider comfort, patient satisfaction with privacy, and patient comfort with audio/visual technology [54] (Cronbach alpha=.76).

#### Knowledge of Genetic Disease (T0-T2)

Participants completed 6 selected items utilized in related research [43,44,55]. This scale included items evaluating cancer inheritance (one item), the meaning of positive results (2 items), and the meaning of negative results (3 items). Internal consistency in this study was good (Cronbach alpha=.62).

#### Psychosocial Adjustment (T0-T2)

Psychosocial adjustment was evaluated with the following three measures (T0-T2):

1. State anxiety was measured with the 20-item State Inventory of the State-Trait Anxiety Inventory (Cronbach alpha=.96) [56,57].

2. General anxiety and depression were assessed with the Hospital Anxiety and Depression Scale (HADS), anxiety and depression subscales (Cronbach alpha=.86 and .84, respectively) [58,59].

3. Cancer worry was evaluated with the Impact of Events Scale (Cronbach alpha=.89) [60,61].

### Statistical Analyses

We used descriptive statistics to describe participant and nonparticipant characteristics. Our primary outcome was feasibility, defined as both adequate uptake (eg, patient willingness) and successful completion of telegenetic visits. Adequate uptake was defined as at least 50% of patients agreeing to RVC telegenetic visits and at least 50% of those who proceeded with testing agreeing to receive their results by RVC. The decision rule was determined to provide sufficient power. With promising uptake and proceeding rates of 60% each, we would have 93% power to declare a future study feasible. With discouraging uptake and proceeding rates of 45%, we would have a 4.7% type I error rate of declaring a future study feasible. The power and type I error rates were calculated using exact binomial inference. We calculated means, standard deviations, and proportions for all constructs in the dataset and evaluated changes in theoretically informed secondary outcomes from baseline to after pretest counseling, and baseline to postdisclosure of test results. We used Fisher’s exact tests, paired *t* tests, and simple linear regressions for hypothesis testing. *P* values of less than .05 based on two-sided hypothesis tests were considered statistically significant.

Framework analysis was utilized to analyze open-ended responses [62,63]. Two research staff members (DH and ES) independently reviewed responses, utilizing thematic analysis to record primary and secondary themes for each item. Disagreements in coding assignments were resolved by a third reviewer (AB).

## Results

### Participant Characteristics

Participant characteristics are described in Table 1. Participants at Bayhealth Medical Center (BMC) in Delaware and Kennedy Health System (KHS) in New Jersey were more likely to be nonwhite and less likely to have graduated college. Patients recruited at BMC were more likely to have had a personal history of cancer. A total of 82 out of 100 (82.0%) approached patients consented to the study (see Figure 1). None reported declining participation in the study due to discomfort with videoconferencing.

### Uptake and Successful Completion of Telegenetic Services

A total of 61 out of 100 (61.0%) approached patients ultimately completed pretest counseling (see Figure 1). There were no differences between those who did and did not complete pretest counseling. A total of 41 of 61 (67%) patients who completed pretest counseling proceeded with genetic testing and received results by RVC. Participants who did not proceed with testing were either not the best candidate in the family for testing (ie, they were unaffected and another family member was the most informative and better candidate for genetic testing) and/or they did not meet payer criteria for insurance coverage for testing. Patients who proceeded with testing were more likely to have a history of cancer (see Table 1). A total of 4 unrelated patients out of 41 (10%) received a positive genetic test result—2 *BRCA2* carriers, 1 *MSH2* carrier, and 1 *PMS2* carrier.

Among 102 completed RVC visits—61 pretest and 41 post-test—only 4 (3.9%) were aborted due to technology failures (ie, lost connections that could not be resolved with multiple attempts). These were believed to be secondary to severe weather (1/102, 1.0%) or connectivity issues at one of the 2 participating sites (ie, the community site or host site). A total of 2 pretest visits were rescheduled for another day and 2 aborted post-test visits were completed by phone. A total of 31 out of 102 (30.4%) visits had disconnections but were resumed and completed during the scheduled appointment. Pretest and post-test visits lasted an average of 61 minutes (range 22-115) and 25 minutes (range 6-63), respectively.

### Patient-Reported Advantages, Disadvantages, and Satisfaction With Real-Time Videoconferencing Telegenetic Services

As shown in Table 2, the most frequently reported advantages of RVC telegenetic services were reducing the burden of traveling (pretest 31/51, 61%; post-test 22/36, 61%), and the convenience and ease of local services during pretest (23/51, 45%) and post-test (8/36, 22%) visits. Other patient-reported advantages included informational value, efficiency, and the benefit of services in their local and familiar medical facility. The majority of participants reported no disadvantages (pretest 36/46, 78%; post-test 28/35, 80%) and had no recommendations for improvement (pretest 43/47, 91%; post-test 35/36, 97%). Some reported technical challenges and that visits felt less personal.

**Table 2:** Patient-reported advantages and disadvantages of remote telegenetic services.

Coded themes^a^		Representative quotes	After pretest counseling (V1^b^), n (%)	Post- disclosure (V2^c^), n (%)
**What did you like about receiving your GC** ^d^ **by telemedicine?** **(V1 n=51; V2 n=36)** ^e^				
	Reduced travel burden	“Telemedicine made it easier to consider genetic testing. I would not have made the effort to travel to another city for testing.” “I could not have physically traveled to speak to a genetic counselor in person due to my present condition, so for me the telemedicine made genetic counseling possible.”	31 (61)	22 (61)
	Convenience/ease	“I was able to combine with my hospital visit.” “It was easy, convenient, and stress free.”	23 (45)	8 (22)
	Informative	“The genetic counselor was very helpful, informative, and thorough.”	7 (14)	5 (14)
	Efficient	“I didn't have to wait like I would in a doctor's office.”	4 (8)	5 (14)
	Personalized	“I enjoyed the one-on-one session. It felt personal and all about me.”	3 (6)	0 (0)
	Good experience	“It was my first time utilizing telemedicine. It was a good experience.”	2 (4)	4 (11)
	Ability to receive services in local facility	“Being able to receive all information locally with my physician present was much better.”	0 (0)	3 (8)
**What did you dislike about receiving your genetic counseling by telemedicine?** **(V1 n=46; V2 n=32)** ^f^				
	No dislikes		36 (78)	28 (88)
	Technical difficulties	“It was a little hard to hear...my voice would echo so it made it a little difficult to answer the questions.” “There was a tech glitch in the beginning but it was fixed. I was concerned that it wouldn't be resolved.”	8 (17)	3 (9)
	Less personal	“It was strange not being able to make actual eye contact.” “It was uncomfortable and not personable.”	2 (4)	2 (6)
**Is there anything you would have changed about receiving your genetic counseling by telemedicine? (V1 n=47; V2 n=36)** ^g^				
	No changes		43 (91)	35 (97)
	Improve technology	“Better sound and eye contact from the counselor.” “Better technology.”	2 (4)	0 (0)
	Improve visual illustrations	“Make sure the items on the slides are in view.”	3 (6)	1 (3)

^a^Responses could be coded for multiple reasons. Themes reported <2 times are not shown.

^b^V1: visit 1.

^c^V2: visit 2.

^d^GC: genetic counseling.

^e^There were 6 and 5 nonrespondents post-V1 and post-V2, respectively (original V1 n=57; V2 n=41).

^f^There were 11 and 9 nonrespondents post-V1 and post-V2, respectively (original V1 n=57; V2 n=41).

^g^There were 10 and 5 nonrespondents post-V1 and post-V2, respectively (original V1 n=57; V2 n=41).

**Figure 2:**
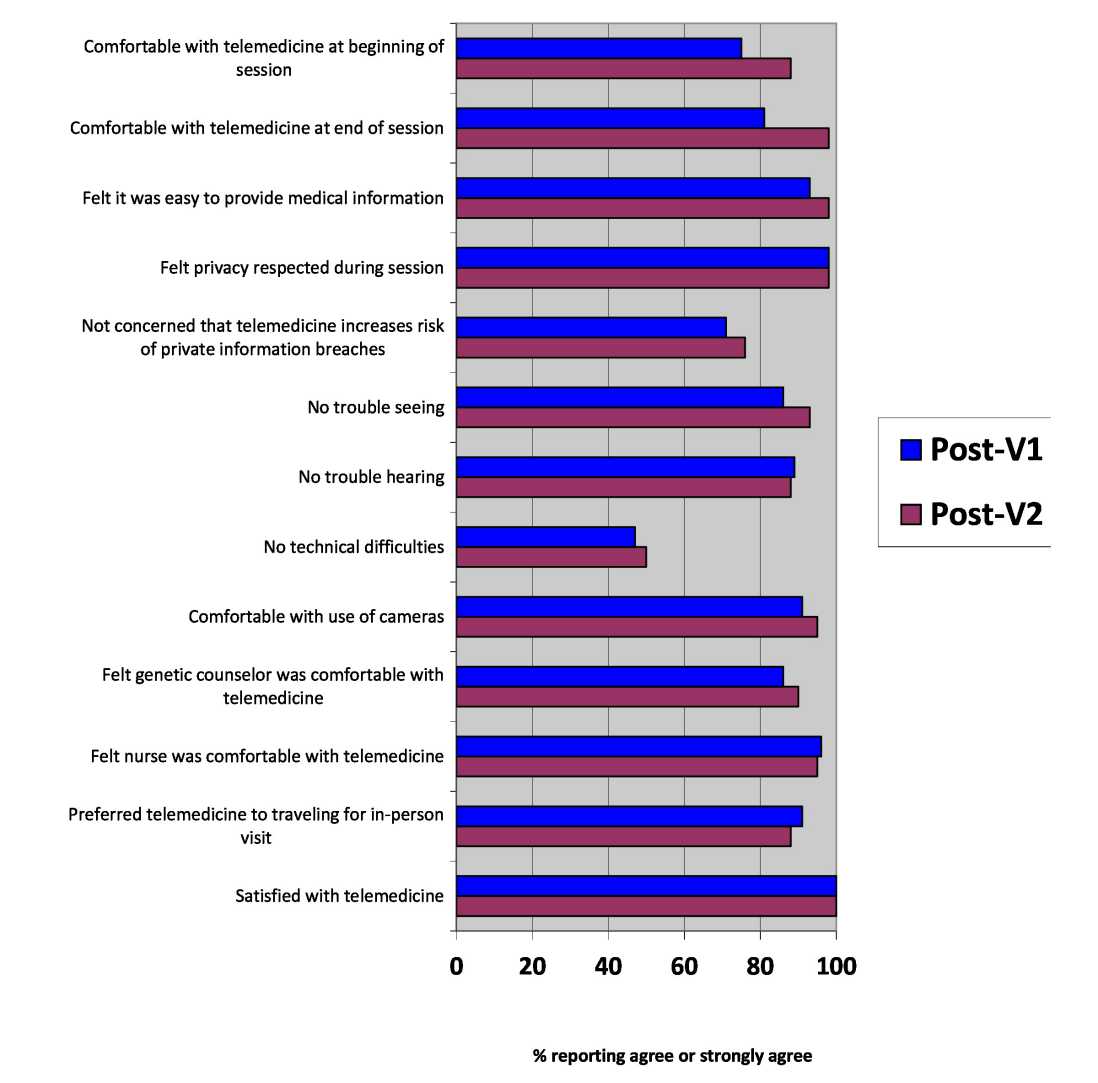
Satisfaction with telemedicine services. V1: visit 1; V2: visit 2.

Patient-reported satisfaction with genetic services and telemedicine services was high, both overall and on specific items (see Figure 2 and Table 3). Most patients reported feeling comfortable with the video camera—post-V1: 52/57 (91%); post-V2: 39/41 (95%)—and that their privacy was respected—post-V1: 56/57 (98%); post-V2: 40/41 (98%)—although some reported concerns that RVC might increase the risk of breach of confidentiality of their health information—post-V1: 14/57 (25%); post-V2: 12/41 (29%). While the majority of patients reported having no trouble seeing or hearing the genetic counselor—post-V1: 47/57 (82%); post-V2: 39/41 (95%)—51 out of 98 (52%) patients reported technical difficulties. Nonetheless, all patients reported being satisfied with genetic services (see Figure 2).

### Cognitive and Affective Outcomes With Telegenetic Services

Among those who completed pretest visits, knowledge increased and general anxiety and depression declined significantly after pretest counseling (see Table 3). State anxiety and cancer worry did not change significantly after pretest visits. Among those who proceeded with genetic testing, satisfaction with genetic services and telemedicine increased significantly after post-test counseling, and depression and anxiety decreased significantly.

**Table 3:** Change in cognitive and affective outcomes with telemedicine delivery of genetic services.^a^

Outcome		Baseline (n=61), mean (SD)	Completed V1^b^ (n=57), mean (SD)	Completed V2^c^ (n=41), mean (SD)	*P*
**General anxiety (range 0-21)**					
	Completed V1	7.34 (4.00)	6.37 (3.99)	N/A^d^	.003
	Completed V1 and V2	6.67 (3.82)	5.59 (3.77)	5.54 (3.50)	.003
**General depression (range 0-21)**					
	Completed V1	3.70 (3.77)	3.33 (3.26)	N/A	.046
	Completed V1 and V2	3.33 (3.43)	3.18 (3.22)	2.58 (3.23)	.01
**Cancer worry (range 0-70)**					
	Completed V1	17.93 (13.06)	16.63 (13.21)	N/A	.36
	Completed V1 and V2	17.10 (13.29)	14.76 (12.06)	16.88 (13.71)	.25
**State anxiety (range 20-80)**					
	Completed V1	37.49 (13.82)	36.49 (12.71)	N/A	.32
	Completed V1 and V2	35.32 (12.97)	34.42 (12.26)	33.29 (11.10)	.27
**Knowledge (range 6-28)**					
	Completed V1	20.96 (2.74)	22.07 (2.99)	N/A	.005
	Completed V1 and V2	21.10 (3.16)	22.14 (3.16)	21.61 (3.16)	.08
Satisfaction with genetic services (range 9-45)					
	Completed V1 and V2	N/A	40.36 (3.92)	42.58 (3.25)	.001
Satisfaction with telemedicine (range 13-65)					
	Completed V1 and V2	N/A	52.25 (5.26)	53.99 (4.96)	.02

^a^Paired *t* tests were performed for changes between two time points; linear regression was estimated by generalized estimating equations to compare time trends for three time points. Time was entered via the use of dummy indicators for each time point in the regressions.

^b^V1: visit 1, pretest counseling.

^c^V2: visit 2, test disclosure.

^d^N/A: not applicable.

We also conducted exploratory stepwise regression analyses to evaluate potential patient factors associated with less favorable select outcomes (eg, less gain in knowledge or greater increase in distress). Older age was significantly associated with lower general anxiety (*P*=.01) at baseline. Being white was associated with greater increases in state anxiety (*P*=.02) after pretest counseling. Being nonwhite was associated with greater increases in state anxiety (*P*=.02) and less satisfaction with genetic services (*P*=.01) after receipt of results. Having a graduate education was associated with greater increases in general anxiety (*P*=.001) after pretest counseling and lower satisfaction with genetic services (*P*=.02). Having more relatives with cancer was associated with lower satisfaction with telemedicine services (*P*=.02), but larger increases in knowledge among those who proceeded with testing (*P*=.001).

## Discussion

### Principal Findings

In this study, we evaluated a resource-extending model by providing genetic services entirely remotely at 3 community medical facilities with no options for in-person genetic services; we found that real-time videoconferencing telegenetic services are feasible, identify genetic carriers in community practices, and are associated with high patient satisfaction and favorable cognitive and affective outcomes. Various videoconferencing extension models have been used to provide telegenetic services. In our study, the genetic provider is physically at the host center. Services are provided entirely remotely in the patient’s local medical facility. Although this is a feasibility study without a comparison arm, to our knowledge it is the largest multicenter study—including 3 community sites in 2 US states outside the host site state—to evaluate the feasibility of offering an entirely remote cancer genetics service by RVC at sites where in-person services are not an option. While the only randomized study of RVC versus in-person genetic services suggests lower uptake of counseling and preferences for in-person services among 32% of participants receiving RVC, traveling of providers to remote sites is more costly and is not feasible in most areas [40]. With increasing attention to medical practice plans and metrics, traveling genetic counselors are diminishing in use, leaving many remote sites entirely without access to genetic providers. Thus, providing specialized services entirely remotely, either by RVC or phone, has the potential to further extend the reach of genetic services. This model also includes collaborative local physician care, which maintains local provider-patient relationships while facilitating cancer susceptibility testing with genetic provider expertise.

### Advantages of Real-Time Videoconferencing Telegenetic Services

Consistent with other studies, this delivery model provides several potential advantages to various stakeholders [17,21,32]. Patients with local providers reported less travel time, fewer travel burdens, and increased informational value by remaining in their local settings. Local providers and practices have access to genetic specialists, while maintaining their local patient-provider relationship. Additionally, studies have suggested that nongenetic physicians are more likely to order unnecessary tests, potentially escalating health care costs [7,8]. In our study, some referred patients were not the best candidate in the family for testing and, thus, testing was not recommended. Thus, RVC telegenetic services might reduce unnecessary testing, providing advantages for payers and the health care system. Equally important, pretest counseling with genetic specialists is one way to facilitate informed decision making for genetic testing, which is becoming increasingly important given the increasing range of testing options (ie, targeted vs multiplex) with variable utility and risk for uncertainty [64]. Providing remote access to the limited workforce of genetic specialists is one way to limit the potential risks of genetic testing as we transition from targeted to broader genetic testing.

Although RVC is technically feasible, technical disruptions or challenges were reported by patients in 52% (51/98) of RVC visits and some patients reported concerns about privacy. Nonetheless, patients were highly satisfied with RVC for cancer genetics services. Many patients indicated that they would not have otherwise received genetic counseling or testing were it not for remote delivery. There were increases in patient knowledge, decreases in depression and anxiety, and no increase in state anxiety or cancer worry. Although this feasibility study did not include a comparison arm, these findings are consistent with published outcomes of telephone and in-person genetic counseling and testing [65,66]. Furthermore, all participants received specialized cancer risk assessment, and 4 families with a genetic predisposition to cancer were identified. While there were technology challenges and disconnections, failure rates were low (and may not be worse than reschedule rates in traditional face-to-face clinic settings). Further, despite technology challenges, patients reported high satisfaction with telegenetic communication and services. Thus, RVC telegenetics provides a feasible alternative model to extend genetic services and identify patients at genetic risk for cancer in communities without local access to genetic services.

To date, there are few studies evaluating RVC for remote clinical delivery of specifically genetic services. There has been only one randomized study that compared RVC to in-person services provided by a traveling genetic counselor. This study reported significant cost savings with RVC. This is consistent with the experience in cancer genetics, as cancer genetics programs have significantly reduced the provision of genetic counselors to satellites given costs. Therefore, while some studies have utilized in-person services as the nonrandomized comparison arm, we propose that the appropriate comparison is usual care, which in these communities typically means patients travel to a regional expertise center or receive testing through their local physician without genetic providers. An example of this design is the randomized study by Myers et al where remote RVC delivery of attention deficit hyperactivity disorder (ADHD) therapy provided by ADHD specialists was compared to ADHD care provided by their primary care providers with a single supplementary RVC specialist visit [16]. Given the limited genetic provider workforce and costs, in-person visits with a genetic provider are not likely to be available in these communities and therefore comparison to in-person visits is not a clinically meaningful, feasible, or real-world comparison. Telephone delivery is a potential alternative delivery model in these settings [43,44,65,66]. In contrast to telephone delivery, RVC has the advantage of maintaining visual communication cues and “face-to-face” communication. To our knowledge, there are no published studies comparing telephone to RVC telegenetic services, but such studies would be valuable to identify the optimal resource-extending model for populations without access to genetic providers. Additionally, given a limited genetic provider workforce, additional models (eg, triaging patients and/or utilizing alternative providers) may be beneficial, but would benefit from evaluation of cognitive, affective, behavioral, and medical outcomes.

### Limitations

We acknowledge several limitations to this study. These centers and patients may be early adopters and larger studies are needed. The number of participants remains relatively small and differences by patient factors (eg, race/ethnicity and education) need to be confirmed in larger studies. Our providers utilized stakeholder-informed communication protocols with training for videoconferencing communication. Outcomes could differ without these features. There was no comparison arm and we cannot comment on the value of RVC telegenetics compared to usual care or telephone delivery, and provider experiences were not assessed. While risk reduction and prevention recommendations presented by the genetic counselor in RVC post-test counseling were reviewed with a physician with expertise in cancer genetics and cancer prevention, in this collaborative telegenetics model post-test medical follow-up occurred with local providers. The uptake of important risk reduction and prevention behaviors (eg, prophylactic oophorectomy and breast magnetic resonance imaging [MRI]) in this delivery model is not yet known. Importantly, there remain many practical challenges to implementing RVC services both in and beyond telegenetics. While many third-party payers do pay for telemedicine services, it can vary by payer and state and has not been tested for genetic counseling [67-69]. Nonetheless, barriers to billing for telegenetic visits may be secondary to the challenges of billing for genetic counseling in general, rather than billing for telehealth; further data regarding payer willingness to pay and reimbursement will be useful. While technology costs are dropping quickly, there are technology costs that could impact the cost and benefit comparison to other remote delivery models (eg, telephone). We utilized a high-quality platform with real-time technical support. Patient and provider experiences could be different with different technology platforms or if extended to the home [39,70]. Lastly, using videoconferencing services and other electronic means of communicating with patients residing in other states may qualify as the practice of medicine in that state, particularly if care has not already been established with a face-to-face visit (ie, providing a new service versus follow-up care). Thus, providers may need to obtain licensure in the state where the patient is located [67]. Additionally, if RVC telegenetic services (ie, phone or videoconferencing) are being provided to inpatients in another state, there may be hospital-credentialing requirements. The legal landscape and the requirements are variable depending on the type of service provided and continue to evolve as new technology evolves. Therefore, until remote services become standard or unified regulations are in place, legal review and oversight is encouraged to ensure compliance with state medical practice laws.

### Conclusions

With expanding testing options in inherited cancer genetics, there is an increasing need to provide access to genetic providers and pretest counseling. Remote real-time videoconferencing is feasible, identifies genetic carriers in community practices, and is associated with high patient satisfaction and favorable cognitive and affective outcomes. Remote videoconferencing provides an innovative delivery model for further study in community practices that lack access to genetic providers, providing the potential to help realize the benefits of genetic medicine across sociodemographically diverse populations.

## Abbreviations

ADHD: attention deficit hyperactivity disorder
BMC: Bayhealth Medical Center
DE: Delaware
FDR: first-degree relative
GC: genetic counseling
HADS: Hospital Anxiety and Depression Scale
IRB: Institutional Review Board
KHS: Kennedy Health System
MAGPI: Mid-Atlantic Gigapop in Philadelphia for Internet 2
MRI: magnetic resonance imaging
N/A: not applicable
NCCN: National Comprehensive Cancer Network
NJ: New Jersey
RVC: remote videoconferencing
SDR: second-degree relative
T0: baseline
T1: pretest
T2: post-test
UPENN: University of Pennsylvania
V1: visit 1
V2: visit 2
VUS: variant of uncertain significance
